# Effect of *Curcuma longa *and *Ocimum sanctum *on myocardial apoptosis in experimentally induced myocardial ischemic-reperfusion injury

**DOI:** 10.1186/1472-6882-6-3

**Published:** 2006-02-19

**Authors:** Ipseeta Mohanty, Dharamvir Singh Arya, Suresh Kumar Gupta

**Affiliations:** 1 Department of Pharmacology, All India Institute of Medical Sciences, Ansari Nagar, New Delhi-110029, India

## Abstract

**Background:**

In the present investigation, the effect of *Curcuma longa *(Cl) and *Ocimum sanctum *(Os) on myocardial apoptosis and cardiac function was studied in an ischemia and reperfusion (I-R) model of myocardial injury.

**Methods:**

Wistar albino rats were divided into four groups and orally fed saline once daily (sham, control IR) or Cl (100 mg/kg; Cl-IR) or Os (75 mg/kg; Os-IR) respectively for 1 month. On the 31^st ^day, in the rats of the control IR, Cl-IR and Os-IR groups LAD occlusion was undertaken for 45 min, and reperfusion was allowed for 1 h. The hemodynamic parameters{mean arterial pressure (MAP), heart rate (HR), left ventricular end-diastolic pressure (LVEDP), left ventricular peak positive (+) LVdP/dt (rate of pressure development) and negative (-) LVdP/dt (rate of pressure decline)} were monitored at pre-set points throughout the experimental duration and subsequently, the animals were sacrificed for immunohistopathological (Bax, Bcl-2 protein expression & TUNEL positivity) and histopathological studies.

**Results:**

Chronic treatment with Cl significantly reduced TUNEL positivity (p < 0.05), Bax protein (p < 0.001) and upregulated Bcl-2 (p < 0.001) expression in comparison to control IR group. In addition, Cl demonstrated mitigating effects on several myocardial injury induced hemodynamic {(+)LVdP/dt, (-) LVdP/dt & LVEDP} and histopathological perturbations. Chronic Os treatment resulted in modest modulation of the hemodynamic alterations (MAP, LVEDP) but failed to demonstrate any significant antiapoptotic effects and prevent the histopathological alterations as compared to control IR group.

**Conclusion:**

In the present study, significant cardioprotection and functional recovery demonstrated by Cl may be attributed to its anti-apoptotic property. In contrast to Os, Cl may attenuate cell death due to apoptosis and prevent the impairment of cardiac performance.

## Background

Recently, the recognition of a different cell death phenomenon 'Apoptosis' has become a major clinical interest. It accounts for a great proportion of cell loss associated with myocardial infarction (MI) and / or ischemia-reperfusion (IR). Cell loss through apoptosis contributes to the impairment of cardiac performance and also plays an important role in myocardial remodeling processes [1,2]. Induction of apoptosis is implicated in myocardial I-R injury among other cardiovascular diseases [3,4]. Various studies have demonstrated that not only reactive oxygen species (ROS) *per se*, but also their oxidation products and other secondary messenger molecules generated by ROS can trigger the programmed cell death [5]. It has been reported that these programmed cell death pathways can be inhibited by antioxidants [6,7] However, there are few studies addressing the inhibition of apoptosis and it's directs on myocardial contractility. Since apoptosis, is a genetically regulated process, hence, a better understanding of the cellular mechanisms that control apoptosis, could lead to defining novel and effective therapeutic strategies to limit the amount of tissue damage in patients with MI [2-4].

*Curcuma longa *(Cl), common Indian dietary pigment and spice has been shown to possess a wide range of therapeutic utilities in the traditional Indian medicine. It's role in wound healing, urinary tract infections, liver ailments are well-documented [8]. The active component of turmeric identified as curcumin exhibits a variety of pharmacological effects including antioxidant, adaptogenic, anti-inflammatory and anti-infectious activities [9,10]. *Ocimum sanctum *(Os), commonly known, as Tulsi in India is a local herb containing potent antioxidants flavanoids (orientin, vicenin) and phenolic compounds (eugenol, cirsilineol, apigenin) [11]. The ancient systems of medicine including Ayurveda, Greek, Roman, Siddha and Unani, have mentioned its therapeutic applications in cardiovascular disorders, diabetes and asthma [12,13]. However, only few studies are presently available that documents its cardioprotective potential. Therefore with the point of view that it might be interesting and possibly fruitful to study the anti-apoptotic properties of *Curcuma longa *and *Ocimum sanctum *(medicinal herbs widely used for the treatment of various diseases in Ayurveda, the Indian System of Medicine) and their effect on ventricular function, the present investigation was planned to unravel the molecular mechanism of the cardioprotective potential of these time tested herbal plants [8-13].

The relative involvement of apoptosis in cardiac I-R was evaluated and the anti-apoptotic activity of these herbs was investigated using immunohistochemical localization of Bax and Bcl-2 proteins and TUNEL staining. To correlate the apoptotic cell death and altered cardiac performance during myocardial I-R changes in the hemodynamic variables were also monitored in the present study. Alterations in MAP, HR, LVEDP, (+) & (-)LVdP/dt were monitored and recorded at preset time points throughout the experimental period (1 hour 45 minutes). Cardioprotective action of these herbal extracts was confirmed by assessing the severity of pathological changes.

## Methods

### Experimental animals

Adult male Wistar rats, 10 to 12 weeks old, weighing 150 to 200 g were used in the study. The study protocol was reviewed and approved by the Institutional Animal Ethics Committee and conforms to the Indian National Science Academy Guidelines for the Use and Care of Experimental Animals in Research. Animals were obtained from the Central Animal Facility of All India Institute of Medical Sciences, New Delhi, India and were maintained under standard laboratory conditions in the department animal house. Rats were housed in polyacrylic cages (38 × 23 × 10 cm) with not more than four animals per cage. They were housed in an air-conditioned room and were kept in standard laboratory conditions under natural light and dark cycles (approximately 14 h light/10 h dark) and maintained at humidity 60 ± 5% and an ambient temperature of 25 ± 2°C. All experiments were performed between 9.0 and 16.0 h. The animals were allowed free excess to standard diet (Ashirwad; Chandigarh) and tap water ad libitum and allowed to acclimatize for one week before the experiments. Commercial pellet diet contained 24% protein, 5% fat, 4% fiber, 55% carbohydrates, 0.6% calcium, 0.3% phosphorous, 10% moisture and 9% ash w/w.

#### Chemicals

All Chemicals were of analytical grade, purchased from Sigma Chemical Co., St Louis, USA. Hydro-alcoholic lyophilized extract of *Ocimum sanctum *was procured from Dabur Research Foundation, and aqueous extract of Curcuma longa was obtained from Sanat Research Laboratories, India. The ABC staining kit and primary (Bax mouse monoclonal IgG_2b _and Bcl-2 mouse monoclonal IgGI) & secondary antibodies (Anti mouse IgG) were procured from Santa Cruz Biotechnology, USA. TUNEL assay kit was purchased from Roche Diagnostics, USA. Double distilled water was used in all biochemical assays.

#### Treatment protocol

The animals were randomly divided into four main groups comprising of thirteen animals each.

#### Group 1 – Saline control group – Sham group

Rats were administered 0.9% normal saline for a month and then sacrificed on the 31^st ^day. The animals were subjected to the entire surgical procedure and thread was passed beneath the coronary artery but the LAD coronary artery was not ligated.

### Pilot study

Cl at the doses of 25, 50, 100 & 200 mg/kg and Os at the doses of 25, 75 & 150 mg/kg were screened in the murine model of isoproterenol induced myocardial necrosis and the optimum dose exhibiting maximum cardioprotective effect was evaluated. Cl (100 mg/kg) and Os (75 mg/kg) doses respectively were found to be the most effective in functional recovery of the heart and favorable restoration of biochemical and histopathological alterations. Hence, these doses were selected for further evaluation singularly as well as in combination in the ischemia and reperfusion model of myocardial injury.

#### Group 2 – Ischemia and reperfusion group – Control IR

In this group, healthy experimental rats were administered 0.9% normal saline for 30 days; thereafter, on the 31^st ^day, the experimental animals were subjected to 45 min LAD coronary artery ligation and 60 min reperfusion induced myocardial injury.

#### Group 3 – Curcuma longa treated group – Cl-IR

Aqueous extract of *Curcuma longa *(100 mg/kg) was administered orally to healthy experimental animals for 1 month. On the 31^st ^day, rats were subjected to 45 min LAD ligation and 60 min reperfusion. The number of animals studied in this group was eleven.

#### Group 4 – Ocimum sanctum treated group – Os-IR

Hydroalcoholic extract of *Ocimum sanctum *(75 mg/kg) was administered orally to healthy experimental animals for 30 days, thereafter, on the 31^st ^day the rats were subjected to a protocol of 45 min LAD ligation and 60 min reperfusion induced myocardial injury. The number of animals studied in this group was thirteen.

The detailed experimental protocol used in the present study is as follows:

### 1) Surgical procedure: infarction protocol and hemodynamic studies

Rats of all the experimental groups were anesthetized intraperitoneally with pentobarbitone sodium (60 mg/kg). Atropine was co-administered with the anesthetic to keep the heart rate elevated especially during the surgery protocol and reduce broncho-tracheal secretions. The body temperature was monitored and maintained at 37°C throughout the experimental protocol. The neck was opened with a ventral midline incision, and a tracheostomy was performed and the rats were ventilated with room air from a positive pressure ventilator (Inco, India) using compressed air at a rate of 70 strokes/min and a tidal volume of 10 ml/kg. The right carotid artery was cannulated and the cannula filled with heparinized saline was connected to the cardiac output monitor CARDIOSYS CO-101 (Experimetria, Hungary) *via *a pressure transducer for measurement of MAP and HR. The left jugular vein was cannulated with polyethylene tube for continuos infusion of 0.9% saline solution. A left thoractomy was performed at the fifth intercostal space and the pericardium was opened to expose the heart. The left anterior descending coronary artery (LAD) was ligated 4–5 mm from its origin by a 5-0 silk suture with a atraumatic needle and ends of this ligature were passed through a small vinyl tube to form a snare. After the completion of the surgical procedure, the heart was returned to its normal position in the thorax. The thoracic cavity was covered with saline-soaked gauze to prevent the heart from drying. The animals were then allowed to stabilize for 15 min before LAD ligation. Myocardial ischemia was induced by one stage occlusion of the LAD by pressing the polyethylene tubing against the ventricular wall and then fixing it in place by clamping the vinyl tube with a hemostat. A wide bore (1.5 mm) sterile metal cannula was inserted into the cavity of the left ventricle from the posterior apical region of the heart. The cannula was connected to a pressure transducer (Gould Stathum P231D) and the whole system was filled with heparinized saline (heparin 50 units/ml). Left ventricular systolic and LVEDP was measured on a multichannel polygraph (Grass 7D, USA) from the left ventricular pressures curve at lower and higher sensitivity of the preamplifier respectively. The maximal rate of rise and fall of left ventricular pressure {peak (+) LVdP/dt and peak (-) LVdP/dt} were measured by the electronic differentiator from the signal output of the channel recording left ventricular pressure. A bolus of heparin (30 IU) was administered immediately before coronary artery occlusion for prophylaxis against thrombus formation around the snare. The animals then underwent 45 min of ischemia, confirmed visually in situ by the appearance of regional epicardial cynosis and ST-segment elevation. The myocardium was reperfused by releasing the snare gently for a period of 60 min. Successful reperfusion was confirmed by visualization of arterial blood flow through the artery, appearance of hyperemia over the surface of the previously ischemia cynotic segment. At the end of reperfusion period, animals were sacrificed for immunohistochemical and histological studies by an overdose of anesthesia.

### 2) Apoptotic studies

#### i) Immunostaining for the localization of Bax and Bcl-2 proteins

A monoclonal mouse anti-human Bcl-2 and Bax proteins as the primary antibody were used for Bcl-2/Bax immunohistochemical staining. The ImmunoCruz Staining Systems utilizes a horseradish peroxidase (HRP)-streptavidin complex for staining of formalin-fixed paraffin-embedded myocardial sections. Indirect immunoperoxidase staining was performed as described with some modifications [27]. Briefly, 4–6 micron thick fixed paraffin tissue sections were subjected to the following immunohistochemical procedure for the localization of Bax and Bcl-2 proteins using specific mouse monoclonal primary antibodies. Sections are first blocked, and then incubated in primary antibody. Biotinylated secondary antibody is added followed by the addition of HRP-Straptavidin complex. The target protein (Bax/Bcl-2) was visualized by incubation in peroxidase substrate (H_2_O_2_) using DAB (3,3' diaminobenzidine) as the chromogen.

#### ii) Terminal Deoxyribonucleotidyl Transferase-Mediated dUTP Nick End Labeling (TUNEL Assay)

Myocardial apoptosis was quantified by detection of DNA fragmentation using the TUNEL technique. Briefly, the enzyme terminal deoxynucleotidyl tranferase was used to incorporate residues of digoxigenin nucleotide into 3' OH ends of DNA fragments. The free end of cellular DNA was labeled by incubating the specimens in streptavidin conjugated to horse radish peroxidase enzyme and peroxidase substrate. The signal of terminal deoxynucleotidyl tranferase-mediated dUTP nick end labeling (TUNEL) assay was used to identify apoptotic cells using secondary reaction with antibodies and DAB chromogen [25]. The slides were counterstained in hematoxylin and total cell counts and TUNEL positive cells in the specimens were determined by means of a light microscope. The cells with clear nuclear labeling were defined as TUNEL positive cells. The apoptotic cells i.e. TUNEL positive cells were expressed as percentage of normal nuclei.

### 3) Histopathological studies

At the end of the experiment, myocardial tissue was immediately fixed in 10% buffered neutral formalin solution. The tissues were carefully embedded in molten paraffin with the help of metallic blocks, covered with flexible plastic moulds and kept under freezing plates to allow the paraffin to solidify. Cross sections (5 μm thick) of the fixed myocardial tissues were cut. These sections were stained with hematoxylin and eosin (H&E) and visualized under light microscope to study the light microscopic architecture of the myocardium. The investigators performing the histologic evaluation were blind to biochemical and hemodynamic results and to treatment allocation. The degree of necrosis was graded and scored as follows:

Score (-): Absence of any inflammation, edema and necrosis

Score (+): Focal areas of inflammation, edema and necrosis

Score (++): Patchy areas of inflammation, edema and necrosis

Score (+++): Confluent areas of inflammation, edema and necrosis

Score (++++): Massive areas of inflammation, edema and necrosis

### Statistical analysis

All numerical data in text, figures and tables are expressed as the mean ± SD. Statistical analysis was performed by one-way analysis of variance (ANOVA) or repeated measures ANOVA when data were compared at different time points within a study group and for time courses between study groups, followed by the Bonferroni post hoc test. Differences were considered statistically significant at p < 0.05.

## Results

### Effects of *Curcuma longa *(Cl) and *Ocimum sanctum *(Os) on

#### 1) Hemodynamic variables following I-R induced myocardial injury

##### i) Mean Arterial Pressure (MAP) and Heart Rate (HR)

The initial value of MAP and HR in the control IR group was 124.7 ± 9.4 mm Hg (Graph 1) and 349 ± 22.6 beats/min (Graph 2) respectively. Both the hemodynamic variables in the control IR group remained depressed throughout the I-R duration as compared to sham baseline values.

In the Cl treated group the baseline values of MAP (Graph 1) and HR (Graph 2) were 126.2 ± 13.9 mm Hg and 339 ± 35.3 beats/min respectively. Cl treatment failed to significantly restore both the hemodynamic parameters as compared to control IR values at similar time points. The value of MAP and HR remained more or less in the same range as observed in the control IR group, during the entire period of LAD coronary artery ligation and reperfusion as compared to control IR group.

The MAP recording in the Os treated group, before LAD coronary artery ligation, was 123 ± 11.9 mm Hg (Graph 1). During the entire ischemic duration, the value of MAP was more or less comparable to control IR recording at similar time points. The MAP showed a gradual decline with the progression of the reperfusion period. However, at 60 min following reperfusion a significant (p < 0.05) correction in the value of this parameter was recorded in the Os treated group as compared to control IR group. The initial value of HR in the Os treated group was 352 ± 25.1 beats/min (Graph 2). Os treatment did not bring about any significant change in HR when compared to control IR values during the entire duration of 45 min of ischemia and 60 min of reperfusion.

##### ii) Left ventricular (+) and (-) maximal rate of change of pressure {(+) & (-) LVdP/dt}

In the control IR group, the (+) LVdP/dt and (-) LVdP/dt values before LAD occlusion was 3140 ± 357 mm Hg/s (Graph 3) and 3004 ± 323 mm Hg/s (Graph 4) respectively. At 5 and 45 min post occlusion there was a significant (p < 0.05) fall in (+)LVdP/dt in the control IR group and reperfusion after 45 min of ischemia also brought about a significant fall in (+) LVdP/dt at 15, 30, 45 (p < 0.05) & 60 (p < 0.001) min compared to sham baseline values. (-)LVdP/dt in the control IR group remained significantly depressed throughout the entire ischemic and reperfusion duration, the maximal fall was recorded at the end of the reperfusion period (p < 0.001) as compared to sham.

The baseline values of (+) & (-) LVdP/dt in the Cl treated group were 3202 ± 157 and 3142 ± 257 mm Hg/s respectively (Graph. 3 & 4). Although there was a steady and continuous fall in the (+) & (-) LVdP/dt in this group during the experimental duration, the value of both these parameters were higher throughout the 45 min of LAD occlusion and 1 h reperfusion period as compared to the control IR group. At 60 min post reperfusion, Cl significantly improved myocardial contractility (p < 0.01) and relaxation (p < 0.05) in comparison to control IR values at same time points.

The baseline values of (+) & (-) LVdP/dt in the Os treated group before LAD coronary artery occlusion was 3184 ± 125 and 2953 ± 165 mm Hg/s respectively (Graph 3 & 4). Os treatment failed to significantly improve both myocardial contractility and relaxation during ischemia and reperfusion duration as compared to control IR values at similar time points.

##### iii) Left Ventricular End Diastolic Pressure (LVEDP)

In the control IR group, following LAD coronary artery occlusion, the LVEDP started rising gradually from its baseline value of 3.3 + 0.91 mm Hg and it remained elevated throughout the ischemic duration as compared to sham (Graph 5). The increase in this variable was most significant (p < 0.001) at 45 min of ischemia. On reperfusion, there was a marked and sudden fall in the value of this parameter during the reperfusion phase, but it remained significantly increased at 5 (p < 0.001) and 15 (p < 0.01) min post reperfusion compared to the sham group.

The baseline value of LVEDP in the Cl treated group was 3.4 ± 0.25 mm Hg (Fig 5). Cl treatment significantly corrected the elevated LVEDP levels at 15 min (p < 0.001), 25 min (p < 0.01), 35 min (p < 0.05) and 45 min (p < 0.001) as compared to control IR values. Thereafter during the reperfusion duration the value of LVEDP in the Cl treated group was comparable to control IR recordings at the similar time points.

The baseline value of LVEDP in the Os treated group was 3.8 + 0.64 mm Hg (Graph 5). After 5 min of coronary artery occlusion LVEDP increased significantly and remained elevated during LAD occlusion period in comparison to sham. However, in the Os treatment group the value of LVEDP was significantly lower at 15 and 45 min of ischemia (p < 0.01) as compared to control IR values at the same time points. Post reperfusion the LVEDP values in the Os treated group were comparable to control IR values.

#### 2) Myocardial apoptotic parameters following I-R induced myocardial injury

##### i) Myocyte Bax protein expression

**Figure 1:**
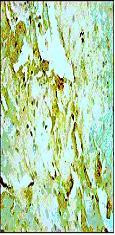
Representative photomicrographs of ventricular tissue stained for Bax protein. Ischemia and reperfusion induced myocardial injury significantly increased the expression of Bax protein compared with non-ischemic tissue. Figures are representative of 5 separate experiments.

**Figure 2:**
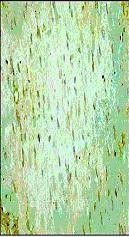
*Curcuma longa *(100 mg/kg) significantly attenuated the expression of Bax protein versus the control IR group.

**Figure 3:**
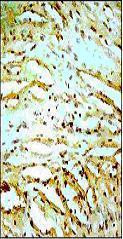
*Ocimum sanctum *(75 mg/kg) treatment did not result in significant alteration in the expression of Bax protein versus the control IR group.

Slight Bax immunoreactivity (3.5% ± 0.4%) was observed in the myocytes of the sham group (Table 1). Ischemia and reperfusion induced myocardial injury significantly increased the expression of Bax protein (p < 0.001) compared with non-ischemic tissue from 3.50 ± 0.40 to 9.80 ± 0.50% (Fig 1). Bax expression was significantly attenuated to 4.4 ± 0.28% and 6.4 ± 0.62% respectively in the Cl-IR (p < 0.01, Fig 2) and Os-IR (p < 0.05, Fig. 3) treated groups as compared to control IR.

**Table 1:** Changes in Bax, Bcl-2 protein expression and TUNEL positivity in the different experimental groups

	**Bax (%)**	**Bcl-2 (%)**	**TUNEL (%)**
**Sham**	3.5% ± 0.4	1.86 ± 0.17	0.2 ± 0.01
**Control IR**	9.80 ± 0.50^###^	1.45 ± 0.21	3.0 ± 0.20^###^
**Cl-IR**	4.4 ± 0.28**	7.7 + 0.62***	0.8 ± 0.04*
**Os-IR**	6.4 ± 0.62*	1.80 ± 0.07	2.4 ± 0.09

Each value is expressed as Mean ± SD of eight experiments. ###p < 0.001 Vs Sham; *p < 0.05, **p < 0.01, ***p < 0.0001 Vs Control IR. Total cell counts and Bax, Bcl-2 and TUNEL positive cells in the specimens were determined by means of a light microscope. The Bax, Bcl-2 and TUNEL positive cells were expressed as percentage of normal nuclei.

##### ii) Myocyte Bcl-2 protein expression

**Figure 4:**
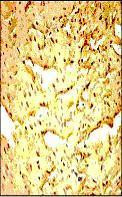
Representative photomicrographs of ventricular tissue stained for Bcl-2 protein. Coronary occlusion and reperfusion resulted in a slight reduction (non significant) in Bcl-2 expression in the control IR group compared to non-ischemic tissue. Figures are representative of 5 separate experiments.

**Figure 5:**
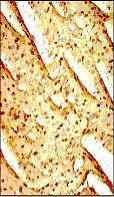
*Curcuma longa *(100 mg/kg) upregulated Bcl-2 protein as indicated by dark brown positive immunoreactivity compared to control IR group.

**Figure 6:**
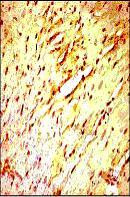
*Ocimum sanctum *(75 mg/kg) treated group subjected to 45 min of ischemia and 1 h of reperfusion failed to demonstrate any significant change in the expression of Bcl-2 protein versus the control IR group.

Bcl-2 protein was expressed in the sham myocardium as indicated by slight positive Bcl-2 immunoreactivity in the myocytes. The basal expression of Bcl-2 was found to be 1.86 ± 0.17% (Table 1). Coronary occlusion and reperfusion resulted in a slight reduction (non-significant) in Bcl-2 expression compared with non-ischemic tissue (Fig. 4). Significant upregulation in the expression of Bcl-2 was observed in the Cl (p < 0.001) treated group in comparison to control IR (Fig. 5). However, in the Os (75 mg/kg, Fig. 6) group there was no significant change in the expression of Bcl-2 protein and it was found to be comparable to control IR group i.e. 1.80 ± 0.07%.

##### iii) TUNEL positivity

**Figure 7:**
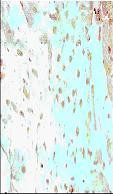
Representative photomicrographs of ventricular tissue stained for nick-end labeling (TUNEL) for DNA breaks. In the Control IR group a large number of TUNEL positive cells are observed subsequent to ischemia and reperfusion injury. Figures are representative of atleast 5 separate experiments.

**Figure 8:**
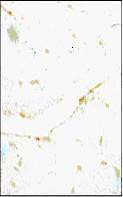
Relative to the control IR group the number of TUNEL positive cells was significantly decreased by treatment with *Curcuma longa *(100 mg/kg).

**Figure 9:**
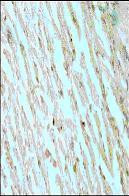
*Ocimum sanctum *(75 mg/kg) treated group subjected to 45 min of ischemia and 1 h of reperfusion failed to significantly decrease TUNEL positivity as compared to control IR group.

TUNEL positivity was expressed as percentage of total normal nuclei. Slight TUNEL positive staining was detected in the sham group (0.2% ± 0.01%, Table 1). However, the number of TUNEL positive cells expressed as percentage of total normal nuclei was significantly increased subsequent to ischemia and reperfusion induced myocardial injury in the control IR group (3.0 ± 0.2%, p < 0.001, Fig. 7) compared to sham non-ischemic myocardium as indicated by increased intensity of TUNEL staining. The TUNEL positivity was significantly attenuated to 0.8 ± 0.04% in the Cl-IR (p < 0.05, Fig. 8) group as compared to control IR. However, Os (75 mg/kg, Fig. 9) treated group did not demonstrate any significant anti-apoptotic activity in comparison to control IR group. The percentage of TUNEL positive cells in the Os-IR was found to be 2.4 ± 0.09%.

#### 3) Myocardial histology and histopathology following I-R induced myocardial injury

Microscopic histology revealed that the non-infarcted myocardium in the sham group was characterized by an organized pattern and shows normal architecture of the myocardium (Table 2). Contrastively, on histological evaluation, rat hearts, subjected to I-R (Control IR) demonstrated marked edema, confluent areas of myonecrosis, myofiber loss and mild inflammation as compared to those in the sham group (Table 2). Cl (100 mg/kg) treated rats demonstrated marked structural improvement specially with regard to the degree of myonecrosis, infiltration of inflammatory cells and edema as compared to the control IR group. However, Os (75 mg/kg) treatment failed to preserve the myocardial cellular integrity as evidenced by patchy areas of myonecrosis, edema and moderate degree of inflammation in this group, which was comparable to that observed in control IR group (Table 2).

**Table 2:** Light microscopic changes observed in the different experimental groups

	**Necrosis**	**Edema**	**Inflammation**
**Sham**	-	-	-
**Control IR**	++	+++	+
**Cl-IR**	+	++	+
**Os-IR**	++	++	+

The relative pathological changes are ranked from - = No change; + = Focal change; ++ = Patchy change; +++ = Confluent change and ++++ = Massive change.

## Discussion

Prolonged severe ischemia leads to cardiac cell death. Necrosis previously was regarded as the only mode of cell death, whereas, now there is accumulating evidence that in addition to overt necrosis, a subset of cells also die by apoptosis, (the programmed cell death). The relative contributions of necrosis and apoptosis to cell death in ischemia and reperfusion are still open to debate, although necrosis appears to dominate during ischemia, and apoptosis may dominate during reperfusion. There is now evidence that apoptosis occurs during sustained ischemia and when reperfusion follows shorter periods of ischemia [14,15].

The progressive loss of cardiomyocytes in a heart that is already compromised leads to further deterioration of cardiac function, conduction disturbances due to degeneration of SA, AV and inter-nodal pathway, cardiac remodeling and cardiomyopathy [3,4]. The fact that apoptosis plays a role in the tissue damage seen after myocardial infarction has pathological and therapeutic implications. If indeed cardiomyocyte apoptosis plays an important role in initiation and progression of cardiac diseases, drugs that effectively and specifically inhibit apoptosis might be useful therapeutic agents for attenuating myocardial injury due to I-R [16].

In reperfused ischemic hearts increase in oxidative stress, and decrease in antioxidant defense has been reported to lead to cardiac dysfunction partly due to apoptosis [4,17]. However, whether or not plant derived agents, known to possess antioxidant activity may reduce myocardial apoptosis induced by I-R, and thus improve ventricular function and thus attenuate MI has not been directly investigated.

In the present study, TUNEL positivity and the immunohistochemical localization of Bax, an inducer of apoptosis and Bcl-2 proteins, inhibitors of apoptosis were studied to delineate the involvement of apoptosis in I-R induced injury. In order to correlate whether the inhibition of apoptosis has any direct effect on cardiac function, hemodynamic function was also monitored and recorded at preset time points throughout the experimental period. Cardioprotective activity of these herbs was confirmed by assessing the severity of pathological changes.

Results of the present study demonstrated that in the control IR group myocardial I-R injury triggered apoptotic cell death. Increase in TUNEL staining observed in the control IR group suggests a role of apoptosis in contribution of myocardial injury following I-R. A slight reduction in Bcl-2 expression and significant increase in Bax expression as compared with sham group was observed in the control IR group. This observation receives support from earlier studies [18,19]. In addition, consistent with increase in apoptotic cell death, post ischemic reperfusion injury also resulted in significant depression of left ventricular dynamics, peripheral hemodynamics (MAP) and HR.

Cl treatment significantly reduced the percentage of TUNEL positive cells Vs the control IR group, demonstrating its significant anti-apoptotic activity. Treatment with Cl was associated with greater Bcl-2 and attenuated Bax expression as compared to the control IR group. Curcumin, the active ingredient of the rhizome of the turmeric plant (*Curcuma longa*), a commonly used spice, has been reported to prevent cancer in animal tumor models possibly by its apoptosis-inducing and antiproliferative influences [20-22]. However, in the present study, in contrast to earlier reports, marked anti-apoptotic activity of Cl was observed.

Previously we reported that cardioprotective effect of Cl results from the suppression of oxidative stress and correlates with the improved ventricular function [23]. However, till date there are no *in vivo *studies, which have explored the relationship between the putative anti-apoptotic effects of Cl on the functional recovery of ischemia reperfused myocardium. The present study demonstrates that Cl treatment resulted in preserved left ventricular function as reflected by a significant increase in the indices of contractility (+) LVdP/dt, relaxation (-) LVdP/dt and decrease in preload (LVEDP). It is speculated that, Cl treatment may have indirectly restored blood flow in the ischemic regions towards normal as assessed by its efficacy in improving cardiac performance, especially correcting the ischemia and reperfusion-induced increase in LVEDP. However, Cl did not significantly affect MAP and HR. The anti-apoptotic effect of Cl, improved ventricular functions with improved histologic features suggests that treatment with this agent may exert cardioprotective effects following coronary ligation and reperfusion.

Os treatment did not demonstrate any significant anti-apoptotic activity as determined by TUNEL staining and immunohistochemical results. No significant change in the expression of Bax and Bcl-2 proteins was observed with Os treatment as compared to control IR in the present study. It markedly increased MAP, HR and significantly reduced the surrogate preload marker LVEDP as compared to control IR. However, it failed to significantly improve the left ventricular contractility and relaxation and failed to significantly modulate the histopathologic alterations compared to control IR group. Results of the present study demonstrate that Os does not possess significant cardioprotective effects.

The relationship between the possible anti-apoptotic effects of Cl on the functional recovery of ischemic reperfusion injury of the heart has been well elucidated by the dosing protocol of the present study. The exact mechanism by which Cl may reduce myocardial ischemia and reperfusion induced myocardial apoptosis is far from clear presently. Recently it has been reported that Curcumin, an active principle of Curcuma longa reduces cardiomyocytic apoptosis. Curcumin an inhibitor of NF-kappaB, ameliorated the surge of pro-inflammatory cytokines during cardiopulmonary bypass (CBP) and decreased the occurrence of cardiomyocytic apoptosis after global cardiac ischemia/reperfusion injury. The authors proposed that by inhibiting NF-kappaB activation, the up-regulation of cardiac proinflammatory genes can be ameliorated, and the activation of matrix metalloproteinase can be decreased during CPB, thereby lessening severity of cardiac mechanical dysfunction after global cardiac ischemia/reperfusion injury [24,25]. However, in the present study, we have used the aqueous extract of Cl and have proposed a different mechanism for its antiapoptotic effect and correlated it with the ventricular function. Based on the present findings it can be speculated that Cl may attenuate apoptosis via a number of mechanisms: Upregulation of Bcl-2 may result in formation of heterodimers with Bax, resulting in no/fewer free Bax protein available for homodimerization. If Bax homodimers predominate cell death will occur, but when Bcl-2 and Bax heterodimererization prevails cells can survive. Substantial evidence indicates that the mitochondria play a critical regulatory role in the signal transduction pathway leading to apoptosis [4,26]. Loss of contractile cells in the heart poses an additional workload on the remaining viable myocytes that may be unbearable, resulting in pathologic stimuli and death signals. In the present study, In contrast to Os, Cl treatment may have salvaged these myocytes and prevented cell loss induced by apoptosis. Histopathologic evaluation further confirms the cardioprotective potential of such a treatment.

In order to elucidate, the additional mechanisms by which Cl may reduce myocardial apoptosis and the potential clinical implications of such actions, we need to further investigate the relationship of the detrimental effects of key oxidants and apoptotic signals with reperfusion injury. This will lead to a better understanding of basic physiological and pathological mechanisms relevant to myocardial ischemia and reperfusion injury and give new insight to novel therapeutic targets and strategies for its treatment. However, the present study provides a lead for further exploring other mechanisms contributing to the cardioprotective effect of Cl. Whether the conclusions drawn on the basis of the current data can be extrapolated to clinical setting, remains to be defined by well-controlled studies in-patients. Nonetheless, the results of the present study are rather encouraging, because they could unravel a new therapeutic approach for the prevention and/or treatment of ischemic heart disease.

## Conclusion

In the present investigation it was observed that subsequent to ischemia and reperfusion injury, *Curcuma longa *treated group demonstrated significant anti-apoptotic property, which might contribute to the observed preservation in cardiac function and cardioprotective effects. Furthermore, the myocardial salvaging effects of *Curcuma longa *were supported by histopathological studies. In contrast, *Ocimum sanctum *did not exhibit any significant anti-apoptotic effects and cardioprotection.

## Abbreviations

*Curcuma longa *(Cl), *Ocimum sanctum *(Os), ischemia and reperfusion (I-R), mean arterial pressure (MAP), heart rate (HR), left ventricular end-diastolic pressure (LVEDP), left ventricular peak positive (+) LVdP/dt (rate of pressure development) and negative (-) LVdP/dt (rate of pressure decline) and cardiopulmonary bypass (CBP).

## Competing interests

The author(s) declare that they have no competing interests.

## Authors' contributions

IM carried out the experimental work, participated in the sequence alignment, drafted the manuscript and performed the statistical analysis. SKG conceived the study, and participated in its design and coordination. DSA participated in the design of the study and helped to draft the manuscript. All authors read and approved the final manuscript.

## Pre-publication history

The pre-publication history for this paper can be accessed here:



## Supplementary Material

Additional file 1 Time course of changes in MAP in different groups. Each value is expressed as Mean ± SD of eight experiments. *p < 0.05 Vs Control IR Click here for file
