# Supplementary material for: Effect of Curcuma longa and Ocimum sanctum on myocardial apoptosis in experimentally induced myocardial ischemic-reperfusion injury
Source: BMC Complement Altern Med. 2006 Feb 19;6:3. doi: 10.1186/1472-6882-6-3 (PMC1397864; doi:10.1186/1472-6882-6-3)
Supplement: Additional file 1 — Time course of changes in MAP in different groups. Each value is expressed as Mean ± SD of eight experiments. *p < 0.05 Vs Control IR [file 1472-6882-6-3-S1.doc]

## Graph 1

80

90

100

110

120

130

0

5

15

25

35

45

5

15

30

45

60

**Time (Min)**

**mm Hg**

Control IR

Cl-IR

Os-IR

I

R

*****

## Graph 2

275

300

325

350

375

0

5

15

25

35

45

5

15

30

45

60

**Time (Min)**

**beats/min**

Control IR

Cl-IR

Os-IR

I

R

## Graph 3

2500

2750

3000

3250

3500

0

5

15

25

35

45

5

15

30

45

60

**Time(Min)**

**mm Hg/s**

Control IR

Cl-IR

Os-IR

I

R

******

Each value is expressed as Mean + SD of eight experiments. **p<0.01 Vs Control IR.

## Graph 4

2250

2500

2750

3000

3250

3500

0

5

15

25

35

45

5

15

30

45

60

**Time(Min)**

**mm Hg/s**

Control IR

Cl-IR

Os-IR

I

R

*****

## Graph 5

2

4

6

8

0

5

15

25

35

45

5

15

30

45

60

**Time (Min)**

**mm Hg**

Control IR

Cl-IR

Os-IR

I

R

*******

*******

*******

***

*****

******

******
